# The salivary microbiome is consistent between subjects and resistant to impacts of short-term hospitalization

**DOI:** 10.1038/s41598-017-11427-2

**Published:** 2017-09-08

**Authors:** Damien J. Cabral, Jenna I. Wurster, Myrto E. Flokas, Michail Alevizakos, Michelle Zabat, Benjamin J. Korry, Aislinn D. Rowan, William H. Sano, Nikolaos Andreatos, R. Bobby Ducharme, Philip A. Chan, Eleftherios Mylonakis, Beth Burgwyn Fuchs, Peter Belenky

**Affiliations:** 1 Department of Molecular Microbiology and Immunology, Division of Biology and Medicine, Brown University, Providence, RI 02912 USA; 2 Division of Infectious Diseases, Rhode Island Hospital, Alpert Medical School and Brown University, Providence, RI 02903 USA; 3 Department of Medicine, Brown University, Providence, RI 02903 USA

## Abstract

In recent years, a growing amount of research has begun to focus on the oral microbiome due to its links with health and systemic disease. The oral microbiome has numerous advantages that make it particularly useful for clinical studies, including non-invasive collection, temporal stability, and lower complexity relative to other niches, such as the gut. Despite recent discoveries made in this area, it is unknown how the oral microbiome responds to short-term hospitalization. Previous studies have demonstrated that the gut microbiome is extremely sensitive to short-term hospitalization and that these changes are associated with significant morbidity and mortality. Here, we present a comprehensive pipeline for reliable bedside collection, sequencing, and analysis of the human salivary microbiome. We also develop a novel oral-specific mock community for pipeline validation. Using our methodology, we analyzed the salivary microbiomes of patients before and during hospitalization or azithromycin treatment to profile impacts on this community. Our findings indicate that azithromycin alters the diversity and taxonomic composition of the salivary microbiome; however, we also found that short-term hospitalization does not impact the richness or structure of this community, suggesting that the oral cavity may be less susceptible to dysbiosis during short-term hospitalization.

## Introduction

To date, the majority of human microbiome research has focused on profiling bacterial communities within the gut due to their well-established links with multiple disease states, such as Crohn’s disease and obesity^1–8^. In recent years, the oral microbiome has received an increasing amount of attention due in part to the major role it has been shown to play in human health^9–18^. Specifically, oral microbial diversity appears to be a strong indicator of dental health. For example, children with higher levels of oral microbiome diversity were found to have a decreased risk of developing severe caries^19–21^. Alternatively, decreased oral microbiome diversity is associated with a number of diseased states^20, 22^. In both children and adults, the dental plaques of individuals with caries displayed a lower microbial diversity and an increased relative abundance of *Streptococcus* spp. compared with healthy individuals^21^. Additionally, reduced taxonomic diversity in the subgingival microbiome of smokers is associated with severe periodontal disease^22^. It has also been suggested that the oral microbiome may play a role in oral cancers^23–25^. One study analyzing the salivary microbiome of patients with oral squamous cell carcinoma found that the presence of three species (*Capnocytophaga gingivalis, Prevotella melaninogenica*, and *Streptococcus mitis)* was predictive of cancer in 80% of cases^25^. Therefore, it is evident that the oral microbiome is a major determinant of oral and possibly overall health.

While its link to oral health is well established, studies suggest that the oral microbiome may be important in systemic diseases as well. For example, prior research has suggested that poor oral hygiene increases the risk of community-acquired pneumonia in elderly patients^13, 15^. Due to its proximity to the respiratory tract, it is unsurprising that the oral microbiota plays a role in pulmonary infections. However, links between periodontal pathogens and more systemic diseases have been observed in recent years, suggesting that the oral microbiota may also have broader impacts on human health^9, 26–29^. For example, several bacterial genera typically found in the oral cavity (including *Streptococcus, Veillonella*, and *Neisseria*) have been detected in atherosclerotic plaques, providing a link to cardiovascular disease^30–32^. Additionally, recent studies have suggested a growing link between oral dysbiosis and diabetes^9, 26–28^. Diabetic patients have altered subgingival microbiomes, with a higher prevalence of periodontal pathogens such as *Porphyromonas gingivalis* ^27^. Conversely, there is some evidence that unstable periodontitis worsens glycemic control in diabetic individuals, suggesting a potentially important role in regulating host metabolism^33^. All of these findings suggest that the oral microbiota plays a significant role in human health beyond the oral cavity. While these early studies are intriguing, more research is necessary to deepen our understanding of the links between microbial communities, human health, and medical intervention.

The oral microbiome (specifically the salivary microbiome) presents numerous intrinsic advantages that may allow researchers to rapidly profile changes in community structure and link those changes to human health. First, the salivary microbiome appears to be remarkably consistent between individuals and is considerably less diverse in comparison to the fecal microbiota^34^. This consistency and lower diversity should enable greater sequencing depth of these communities and also simplify downstream statistical analyses^35^. Furthermore, this consistency is crucial in the context of longitudinal studies. Previous literature suggests that the gut microbiome is prone to large shifts in diversity and composition in response to several perturbations, including diet, exercise, and hospitalization^6, 7, 36–39^. Such instability could make it difficult in some cases to discern the causes of microbiome shifts and their relevance to human health in the clinical setting. Using a community that is more temporally consistent such as the salivary microbiome may improve our understanding of how different treatments or perturbations affect the human microbiota. Additionally, while studies suggest significant temporal stability, the salivary microbiome has been shown to be perturbed during various disease states, such as polycystic ovary disease and diabetes^28, 29^. Thus, the combination of the salivary microbiome’s stability in the absence of perturbations and its responsiveness to systemic disease make it an intriguing option for analysis in clinical studies.

An added benefit of using this community is that the collection of saliva samples from human patients is significantly less intrusive than fecal collection in both inpatient and outpatient settings. Saliva samples can be rapidly collected from subjects and immediately preserved, allowing accurate profiling of taxonomic changes in response to different treatments or disease states. This feature makes the oral microbiome particularly promising as a tool for clinical research, as it would allow health professionals and researchers to closely monitor the impacts of treatment on the microbiota. Additionally, this contrasts with other microbial niches such as the gut, which is typically profiled using fecal matter that is collected after a lengthy transit through the gastrointestinal tract. This process induces taxonomic shifts, thereby introducing a confounding factor when determining associations between taxa and various health states^40^.

Lastly, the sensitivity of the gut microbiota to external perturbations in a clinical setting complicates its usage in monitoring the effect of specific pathologies and treatments on these communities. Previous studies have reported that hospitalization itself is capable of inducing large taxonomic shifts within the gut microbiome even in the absence of antibiotic treatment^39, 41–43^. These changes not only increase the risk of hospital-associated infections, but introduce confounding factors that would make it difficult to identify the causes of specific shifts in downstream analyses^38, 39, 41–44^. However, little research has been done on how hospitalization, both short- and long-term, impacts the oral microbiome. Thus, we wanted to track the impacts of short-term hospitalization on the composition and diversity of the salivary microbiome.

Here, we present a collection, extraction, sequencing, and analysis workflow that simplifies profiling the salivary microbiome. To do so, we generated and sequenced a novel mock community containing genomic DNA extracted from known residents of the human oral cavity and saliva. Using this resource, we compared the performance of two commonly sequenced regions of the 16S rRNA gene in the analysis of the salivary microbiome. Using our optimized workflow, we subsequently show that the salivary microbiome of patients undergoing short-term hospitalization is remarkably consistent between individuals. This consistency contrasts sharply with other niches such as the gut, which has been shown in previous studies to display much higher levels of inter-individual variation^34^. Furthermore, our data show that azithromycin treatment alters the diversity and taxonomic composition of the salivary microbiome; conversely, we found that this community is largely unaffected by short-term hospitalization. This contrasts with previous findings that indicate that the gut microbiome is profoundly sensitive to the impacts of hospitalization^39, 42, 43^. These findings suggest that short-term hospitalization may not be associated with significant negative impacts on the health of the oral microbiome.

## Results and Discussion

**Figure 1:**
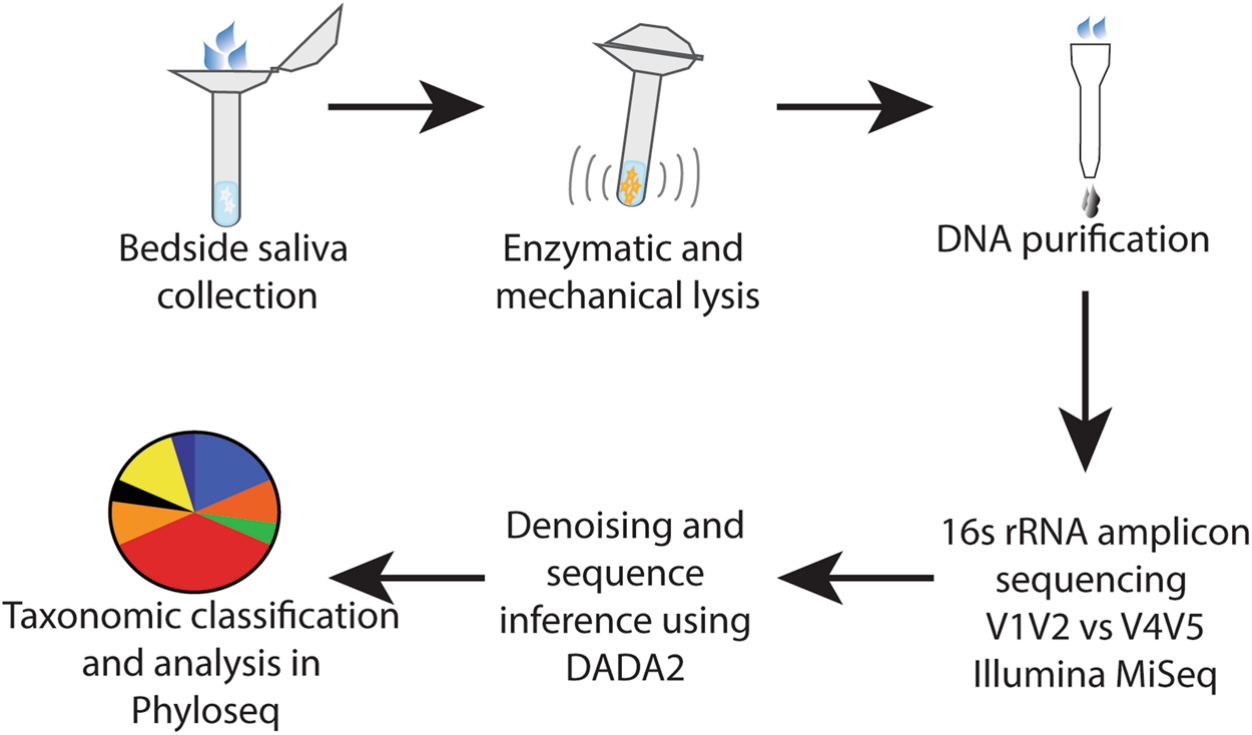
Experimental Workflow. Approximately 1 mL of saliva was collected from study participants using a commercially available kit from OMNIgene. Bacteria within the saliva samples were then vigorously lysed using both enzymatic and mechanical techniques. After purifying genomic DNA, amplicons from the 16S rRNA gene were generated, sequenced on an Illumina MiSeq, and analyzed using the DADA2 computational pipeline to determine microbiome composition.

The primary goal of this study was to identify whether short-term hospitalization impacts the composition of the human salivary microbiota. Before analyzing patient samples, however, it was necessary to develop a comprehensive and streamlined protocol that was capable of effectively and reproducibly profiling salivary communities (Fig. 1). Studies involving the salivary microbiome have sequenced a number of different hypervariable regions using a variety of sequencing platforms^16, 34, 45–48^. Due to a lack of consensus, we chose to develop a methodology pipeline that would allow for convenient systematic collection, sequencing, and analysis for both this study and future work.

### Comparison of Two Regions of the Bacterial 16S rRNA Gene Using a Mock Microbial Community

**Figure 2:**
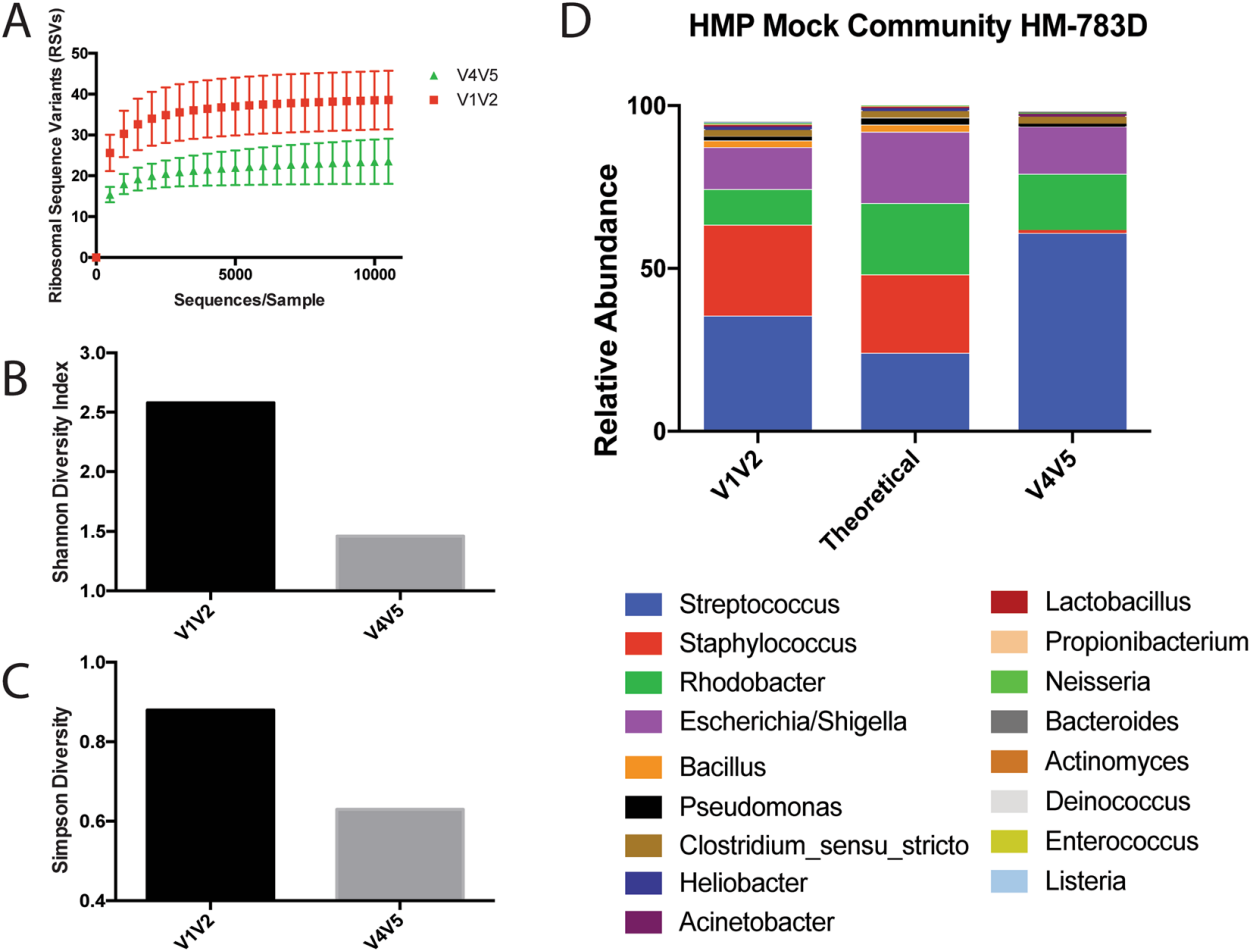
Analysis of Microbial Mock Community HM-783D. (**A**) Rarefaction curves of sequencing data obtained using mock microbial community HM-783D for both V1V2 and V4V5 regions of the 16S rRNA gene. Sequences were subsampled in increments of 500 to an even depth of 14,000 sequences per sample. (**B**) Shannon and (**C**) Simpson diversity calculated for the mock community using the V1V2 and V4V5 hypervariable regions. (**D**) Theoretical and observed relative abundances of genera detected in HM-783D using the V1V2 and V4V5 hypervariable regions.

Before investigating the impact of short-term hospitalization on the salivary microbiome, we sought to identify which region of the 16S rRNA gene was best suited to analyze this community. To do so, we compared the performance of two commonly utilized regions, V1V2 and V4V5, using a mock community composed of bacterial genomic DNA, developed for the Human Microbiome Project (HMP) and available through BEI Resources (HM-783D). The composition of this mock community is summarized in Supplementary Table S1. Rarefaction analysis of the mock community suggested that the V1V2 region can identify more unique ribosomal sequence variants (RSVs) than was possible with V4V5 (Fig. 2A). As a result, the alpha diversity of this mock community was found to be higher using the V1V2 region regardless of which metric was used (Fig. 2B and C). Distinct differences can also be seen between the two regions when comparing relative abundance of taxa at the genus level (Fig. 2D). In total, the V1V2 region could detect 16 of the 17 genera present in the mock community while V4V5 was only able to detect ten (Supplementary Table S2). Furthermore, the V4V5 dataset was dominated by only three genera: *Streptococcus, Escherichia/Shigella*, and *Rhodobacter*. Similarly, the V1V2 dataset was dominated by a small number of genera; using V1V2, however, we could detect *Staphylococcus* - a major component of the HM-783D mock community that was undetected using V4V5 (Fig. 2D). These results are somewhat similar to previous work published by Fouhy *et al*. using an analogous mock community. They also observed that V4V5 was unable to detect *Staphylococcus* in appreciable quantities on an Illumina MiSeq, suggesting that this may be a major limitation of using the V4V5 region in microbiome studies^49^. Therefore, these findings suggest that the V1V2 region is capable of more accurately recapitulating the composition of the mock microbial community.

### Analysis of an Oral-Specific Mock Community

**Figure 3:**
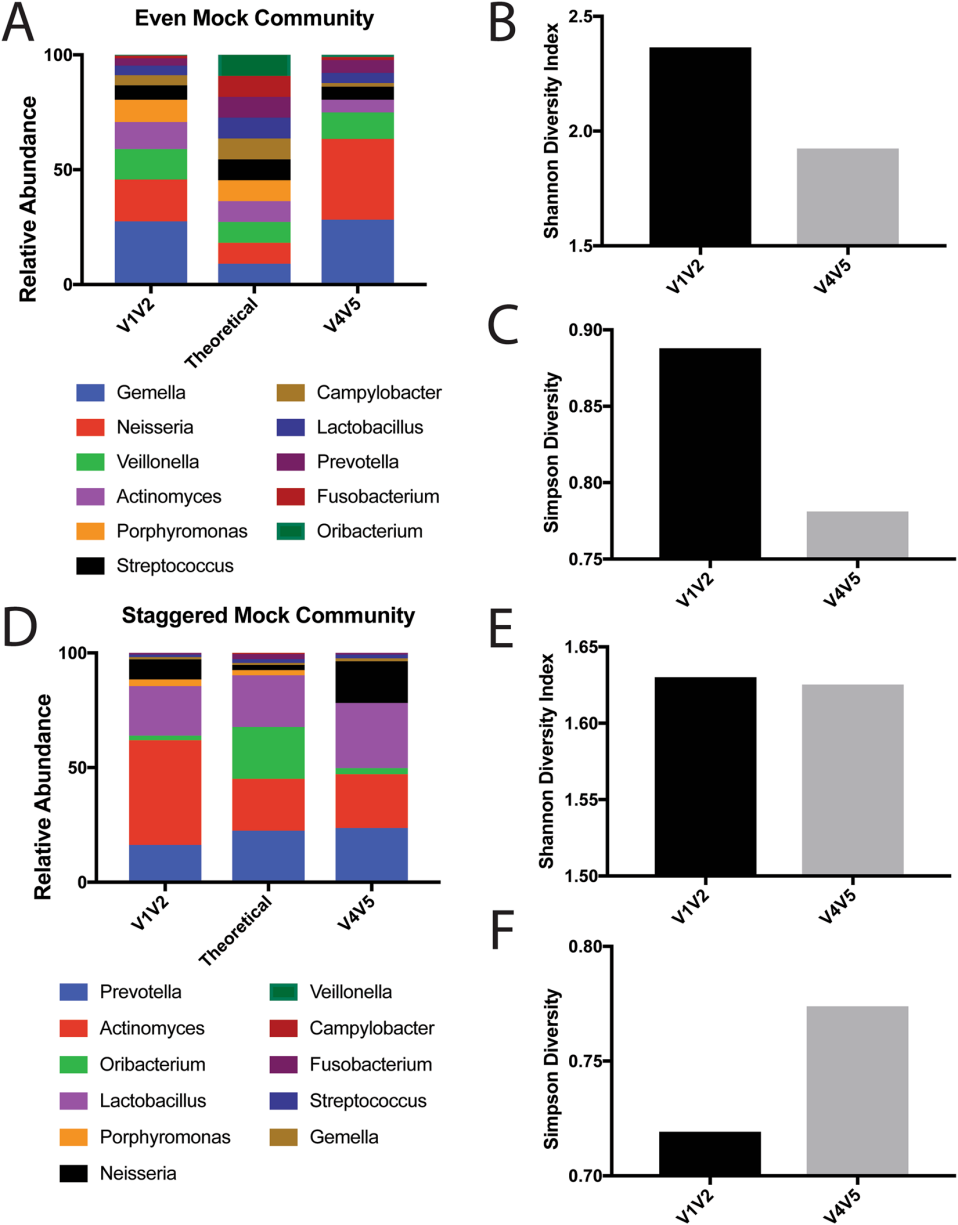
Analysis of a Novel Oral-Specific Mock Community. (**A**) Relative abundances of all eleven phyla present in the even mock community using the V1V2 and V4V5 hypervariable regions. (**B**) Shannon and (**C**) Simpson diversity calculated for the even mock community using the V1V2 and V4V5 hypervariable regions. (**D**) Relative abundances of all eleven phyla present in the staggered mock community using the V1V2 and V4V5 hypervariable regions. (**E**) Shannon and (**F**) Simpson diversity calculated for the staggered mock community using the V1V2 and V4V5 hypervariable regions.

Our preliminary data using the HMP mock community suggested that the V1V2 region can detect more unique taxa within the mock community. However, many of the taxa found in the mock community are not typically found in the oral cavity^34, 50, 51^. Therefore, we generated our own mock communities using genomic DNA extracted from eleven known residents of the oral cavity or closely related species. The composition of these community was based on previously published data and our own preliminary sequencing results. To test the ability of each hypervariable region to detect differences in community composition, we generated both an even and a staggered version of this mock community. The theoretical and observed composition of these mock communities with each region can be found in Supplementary Table S3 and S4, respectively. When analyzing the even mock community, we found that the V1V2 region was able to better capture the true community composition (Fig. 3A). The V4V5 region overrepresented *Neisseria* relative to its true proportions and was nearly unable to detect *Porphyromonas*. While V1V2 somewhat overrepresented *Neisseria*, this effect was much more pronounced with V4V5 (Fig. 3A). We also observed several commonalities between the two regions; for example, both regions overrepresented *Gemella* and underrepresented *Oribacterium* and *Fusobacterium* relative to their true proportions (Fig. 3A). When analyzing alpha diversity, we found that V1V2 was able to detect considerably higher levels of Shannon diversity, a measure of richness (Fig. 3B). Because this community was composed of eleven genera mixed in even proportions, we next analyzed Simpson diversity – a common metric to measure the evenness of a community. We found that the Simpson diversity was considerably higher for the V1V2 region in comparison to the V4V5 region indicating that V1V2 was superior in detecting the evenness of our community and suggesting that V1V2 is better suited to analyze communities of oral bacteria (Fig. 3C).

Next, we analyzed the staggered community and found that both tested regions exhibited distinct strengths and weaknesses. Both regions underrepresented the relative proportions of *Oribacterium* and *Fusobacterium*. However, V1V2 overrepresented *Actinomyces* while V4V5 overrepresented *Gemella* and *Lactobacillus*. As was the case with the even community, both V1V2 and V4V5 overrepresented *Neisseria* relative to its true proportions; however, this effect was more dramatic for V4V5. Similarly, V4V5 was unable to detect *Porphyromonas* in appreciable quantities (Fig. 3D). When we analyzed Shannon diversity, we found that both regions were able to capture approximately the same level of richness within the mock community (Fig. 3E). Interestingly, we found that the V4V5 region detected a higher level of evenness as measured with Simpson diversity (Fig. 3F). However, this finding is problematic because this mock community was not designed to be evenly distributed; therefore, this further suggests that the V4V5 may not accurately represent the true proportions of bacteria typically found in the oral cavity.

### V1V2 is preferred for salivary microbiome analysis

Sequencing of various mock communities suggested that the V1V2 region is superior when analyzing the salivary microbiome. Though our novel mock community contained many of the most abundant taxa typically found in the oral cavity, any mock community is unlikely to completely recapitulate the true diversity found within host-associated microbial communities. Therefore, we wished to test the performance of both regions of the 16S rRNA gene when analyzing saliva samples collected from patients. For our study, we recruited twelve patients that were admitted for treatment to Rhode Island Hospital in Providence, RI. Patients were included in the study if they were over the age of 18 and were excluded if they were receiving immunosuppressive therapy. Additionally, none of the patients had received antibiotics at any point during their participation in this study or in the six months prior to admission; therefore, we could rule out antibiotic-induced perturbations as a confounding factor in our analysis. Each participant provided two saliva samples: one at the time of admission (day 1) and another at three days post-admission (day 4). We chose to perform a 72 hour follow-up because the average hospital stay in the United States is approximately 4.5 days^52^. Lastly, we included patients with a variety of diagnoses in our short-term hospitalization group to account for potential pathology- or treatment-induced effects on the salivary microbiome. Currently, it is largely unknown how the salivary microbiome responds during disease and clinical treatment. Recent work, however, suggests that these communities are altered during diabetes and polycystic ovary disease^28, 29^; therefore, it is likely that other systemic pathologies have a similar impact. By including patients with a wide variety of diagnoses and treatments, we are avoiding bias intrinsic to single pathologies. Otherwise, we concluded that it would be too difficult to determine whether any potential shifts in this community were caused by clinical pathology or by short-term hospitalization itself. However, we excluded all patients receiving antibiotic therapy due to the strong possibility that treatment could cause significant shifts within this community. The characteristics of the participants in our study are summarized in Table 1 along with their respective age, sex, and diagnosis.

**Table 1:** Patients Participating in this Study with Associated Metadata.

Patient ID	Sex	Age	Diagnosis	Antibiotic
P01	Male	67	Bradycardia	None
P02	Female	77	Non-ST elevation myocardial infarction	None
P03	Female	81	Tibial Fracture	None
P04	Male	76	Hypotension	None
P05	Male	67	Aortic Thrombosis	None
P06	Male	64	ST elevation myocardial infarction	None
P07	Male	70	Truncal ataxia	None
P08	Male	56	Watery diarrhea	None
P09	Female	53	Pancreatic cancer	None
P10	Female	84	Hip fracture	None
P11	Female	45	Temporal lobe epilepsy	None
P12	Male	64	Acute pancreatitis	None
P13	Male	27	Chlamydia (rectal)	Azithromycin
P14	Female	20	Chlamydia	Azithromycin
P15	Male	26	Chlamydia	Azithromycin
P16	Male	27	Chlamydia (rectal)	Azithromycin
P17	Female	21	Chlamydia	Azithromycin
P18	Female	19	Chlamydia	Azithromycin
P19	Female	20	Chlamydia (pharyngeal)	Azithromycin
P20	Female	19	Chlamydia	Azithromycin
P21	Male	33	Chlamydia	Azithromycin
P22	Male	52	Chlamydia	Azithromycin
P23	Male	68	Chlamydia (pharyngeal)	Azithromycin
P24	Female	26	Chlamydia	Azithromycin

To collect and store samples, we utilized the OMNIgene-Oral kit (OM-505), which provides a sterile, disposable mouthpiece that reduces both patient inconvenience and the risk of sample contamination (Fig. 1). An added benefit of this kit is that it contains both RNA and DNA stabilization reagents that allow samples to be stored at room temperature for an extended period without appreciable degradation of nucleic acids. Though samples were frozen shortly after collection in our study, this feature would facilitate saliva collection in situations where this may not be possible, such as outpatient studies. Extraction of microbial DNA and amplification and sequencing of 16S rRNA amplicons were performed as described in Materials and Methods.

**Figure 4:**
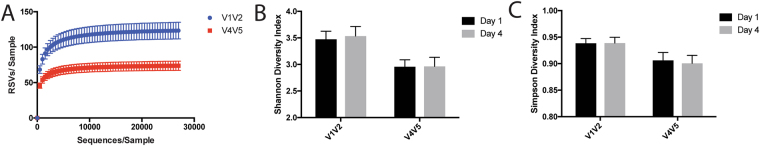
Comparison of Hypervariable Regions for the Analysis of the Alpha Diversity of Saliva Samples. (**A**) Rarefaction curves of sequencing data obtained using saliva samples for both V1V2 and V4V5 regions of the 16S rRNA gene. Sequences were subsampled in increments of 500 to an even depth of 27,000 sequences per sample. (**B**) Average Shannon diversity calculated for saliva samples using V1V2 and V4V5 hypervariable regions on both days of collection. (**C**) Average Simpson diversity calculated for saliva samples using V1V2 and V4V5 hypervariable regions on both days of collection.

**Figure 5:**
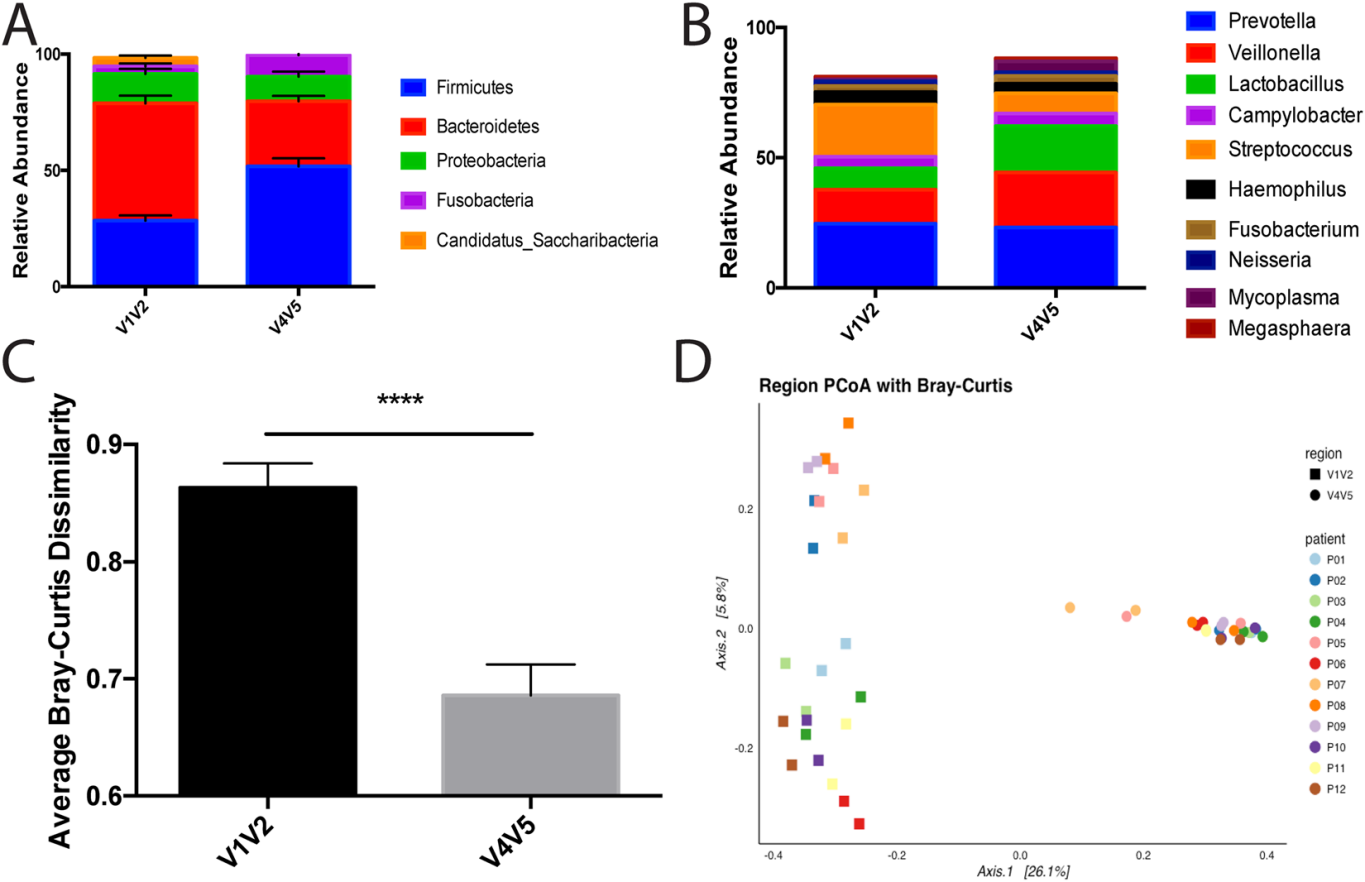
Comparison of Hypervariable Regions for Taxonomic Analysis of Saliva Samples. (**A**) Relative abundances of the top five phyla detected in all saliva samples using the V1V2 and V4V5 hypervariable regions. (**B**) Relative abundances of the top ten genera detected in all saliva samples using the V1V2 and V4V5 hypervariable regions. (**C**) Average Bray-Curtis dissimilarity calculated between patient samples using both V1V2 and V4V5 hypervariable regions. (**D**) Principle Coordinate Analysis (PCoA) of both hypervariable regions using Bray-Curtis dissimilarity.

Rarefaction analysis of human saliva samples demonstrated that the V1V2 region could detect nearly twice as many unique ribosomal sequence variants as V4V5 (Fig. 4A). Thus, the V1V2 samples displayed higher Shannon and Simpson diversities relative to V4V5 – a trend that was consistent for both days of sampling (Fig. 4B and C). It is possible that the elevated diversity could be an artifact caused by sequencing errors. To address this concern, we chose to utilize the DADA2 software package to analyze all of our sequencing data. This package infers sample composition using error rates within a sequencing run and has been shown to produce fewer spurious sequences in comparison to other commonly used analytical pipelines^53^. By using this program, we reduced the likelihood that our rarefaction and alpha diversity analyses would be artificially elevated by sequencing errors. When the taxonomic composition of the samples was analyzed at the phylum level, we detected higher abundances of *Firmicutes* using the V4V5 region while the V1V2 dataset contained a higher abundance of *Bacteroidetes* (Fig. 5A). At the genus level, *Prevotella* was the most abundant taxa detected by both the V1V2 and V4V5 regions (Fig. 5B). However, V1V2 detected higher levels of *Streptococcus* while V4V5 detected comparatively higher levels of *Veillonella* and *Lactobacillus*, two known residents of the oral cavity in humans^19, 21, 24, 26, 30, 45, 50, 54–57^. Together, the higher sequence diversity detected by V1V2 and the known limitations of V4V5 from our mock community analysis suggest that the V1V2 region may be preferable for analysis of the salivary microbiome.

When we compared the beta diversity of both regions, we found that the average Bray-Curtis dissimilarity between samples was significantly higher when using the V1V2 region compared to the V4V5 region (Fig. 5C, unpaired t-test: p < 0.0001). Interestingly, this pattern is reversed when we compared the average weighted and unweighted Unifrac distances (Supplementary Figure S1). Furthermore, principle coordinate analysis (PCoA) using Bray-Curtis Dissimilarity shows that samples cluster according to the region sequenced and that the region sequenced is the primary source of variation between the samples (Fig. 5D). This would also suggest that other factors, such as inter-individual differences and the day of hospitalization, contribute comparatively little to the total variation observed in the data.

Within each cluster, samples from the same patient tended to appear close together, suggesting that there is little temporal variation in the composition of the salivary microbiome regardless of which region is sequenced. We also observed that the samples sequenced using V4V5 in general clustered closer together than those using V1V2. We believe this observation stems from our earlier finding that the V1V2 region can detect more unique ribosomal sequence variants in comparison to V4V5, indicating that V1V2 is more sensitive to differences in community structure between samples. This pattern was similar, though more extreme, when the analysis was repeated with weighted and unweighted Unifrac (Supplementary Figure S1).

Our findings suggest that both regions can be used for the analysis of the salivary microbiome. However, we concluded that V1V2 was noticeably superior to V4V5 for this specific purpose. Analysis of the HMP mock community demonstrated that V1V2 was more effective in detecting members of the genus *Staphylococcus*, an important human commensal^58–60^. Conversely, the V4V5 region appears to allow better detection of the genera *Bacillus* and *Enterococcus*. Because these genera are typically found in soil and the human gut, this would suggest that the V4V5 region may be superior for those sample types instead^41, 58, 61–63^. Additionally, both rarefaction and alpha diversity analysis demonstrated that the V1V2 region is capable of detecting more sequence diversity within saliva samples in comparison to V4V5. For this reason, we suspected that the V4V5 region did not contain enough sequence variation to distinguish as many unique members of this community as did the V1V2 region. Alternatively, the V4V5 primer set could induce biases that result in the preferential amplification of a smaller portion of the microbial community. We conclude that the V1V2 region is better suited for analyzing the salivary microbiome utilizing our sequencing pipeline.

### The salivary microbiome is consistent between individuals and is unaffected by short-term hospitalization

**Figure 6:**
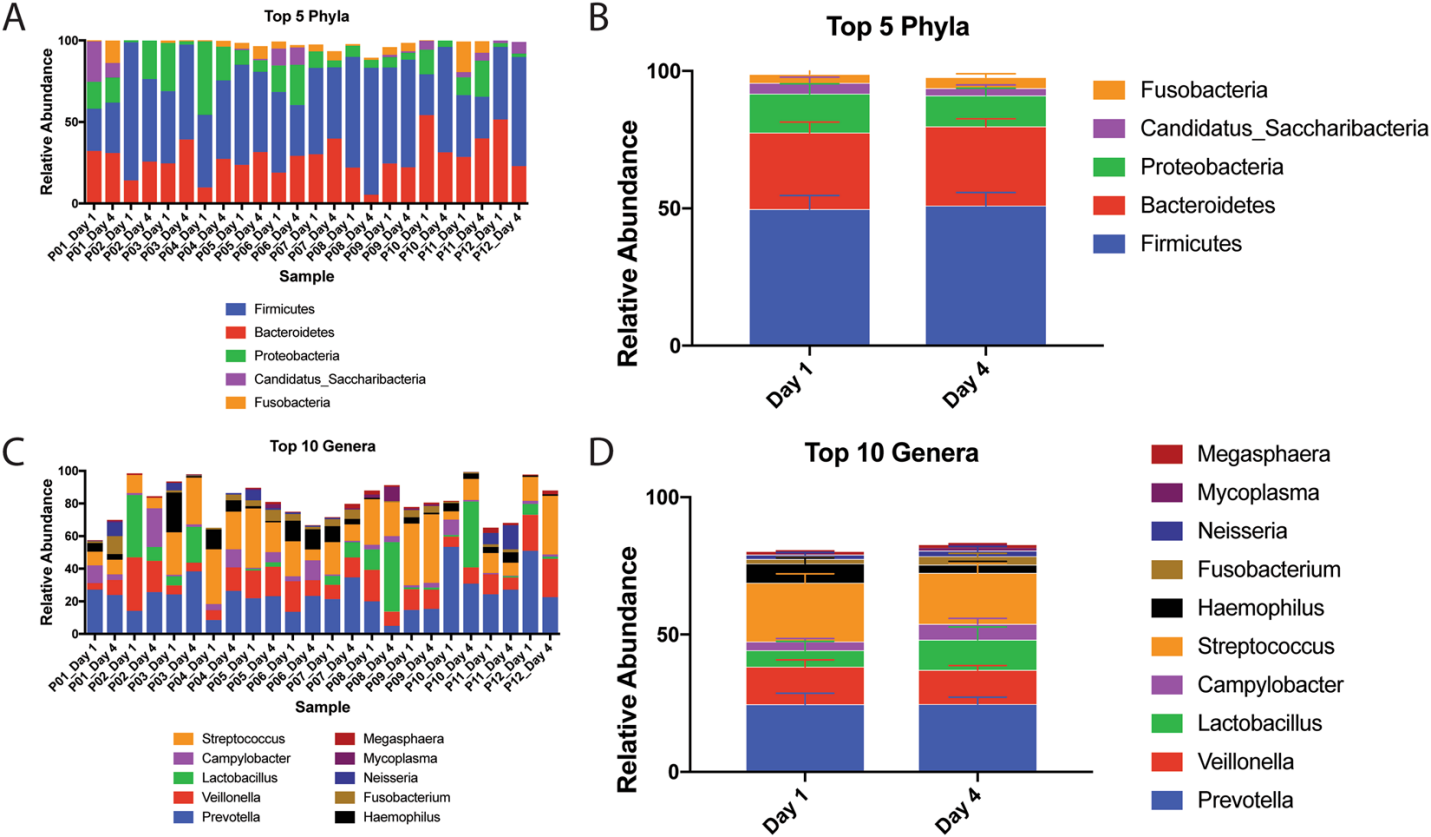
Taxonomic Analysis of Saliva Samples Before and After Hospitalization. (**A**) Relative abundances of the top five phyla detected in each sample using the V1V2 hypervariable region. (**B**) Average relative abundances of the top five detected phyla in hospitalized patient samples on days 1 and 4 of hospitalization. (**C**) Relative abundances of the top genera detected in each sample using the V1V2 hypervariable region. (**D**) Average relative abundances of the top ten detected genera in hospitalized patient samples on days 1 and 4 of hospitalization.

Previous literature has demonstrated that the gut microbiota is exquisitely sensitive to the effects of short- and long-term hospitalization, leading to an increased risk of dysbiosis and opportunistic infections^39, 42–44^. However, it is currently unknown what impact, if any, short-term hospitalization has on the composition of the oral microbiota. Once we had established that the V1V2 region was superior for our analysis, we sought to determine the impact of short-term hospitalization on the composition of the salivary microbiome. In addition, we wanted to examine the inter-individual variation in community composition. When the data were analyzed at the phylum level, we observed that there was little temporal variation in the composition of the salivary microbiome before and after short-term hospitalization (Fig. 6A). Furthermore, we observed that *Firmicutes* was the dominant phylum in nearly every sample, followed by *Bacteroidetes* and *Proteobacteria*. The relative proportions of these phyla varied somewhat between individuals. When all patients are grouped together to account for inter-individual variation, differential abundance testing showed that none of these changes were statistically significant at the genus or phylum level (negative binomial Wald test: p > 0.9) (Fig. 6B and D). Similar results were observed when analyzing the V4V5 dataset (Supplementary Figure S2).

**Figure 7:**
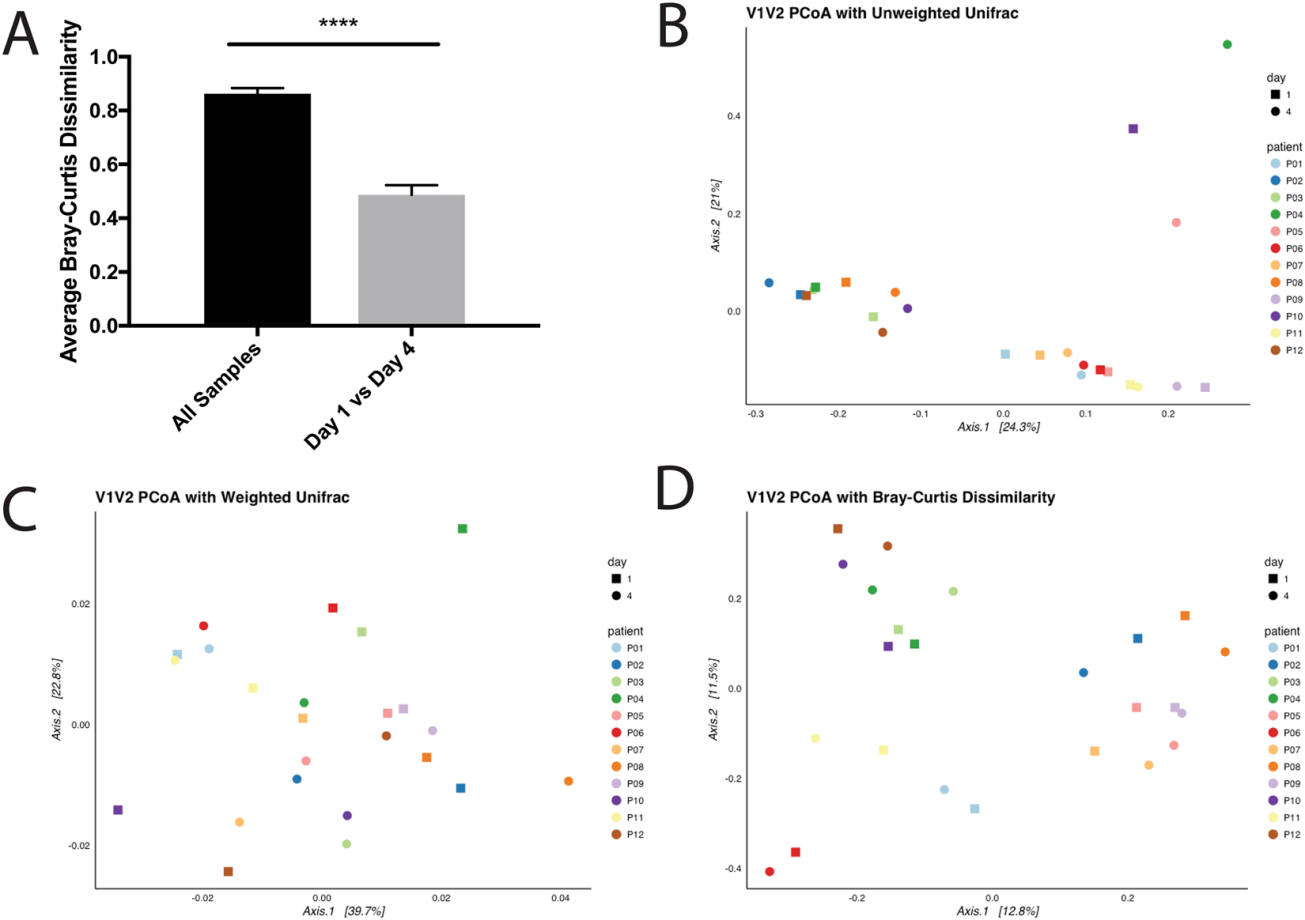
Beta Diversity Analysis of Saliva Samples Before and After Hospitalization. (**A**) Average Bray-Curtis dissimilarity distances between all samples and between sample pairs before and after hospitalization (**B–D**) Principle Coordinate Analyses (PCoA) using unweighted Unifrac, weighted Unifrac and Bray-Curtis dissimilarity for patient samples on days 1 and 4.

To further analyze the changes in community composition associated with short-term hospitalization, we calculated various metrics of alpha and beta diversity. Regardless of which region was analyzed, we did not detect any significant changes in either Shannon or Simpson diversity after three days of hospitalization (Fig. 4B and C), suggesting that short-term hospitalization does not drastically alter the richness of the salivary microbiome. Next, we wanted to determine the impact of short-term hospitalization on the beta diversity of saliva samples. We found that the Bray-Curtis dissimilarity between samples taken from the same individual on two separate days was significantly lower than the average distance between all samples (Fig. 7A, p value < 0.001, unpaired t-test). This finding demonstrates that the distance between samples from the same individual before and during hospitalization is smaller than the distance between any two random individuals, further demonstrating that short-term hospitalization does not contribute to a significant shift in beta diversity. Lastly, we conducted PCoA analyses using unweighted Unifrac, weighted Unifrac, and Bray-Curtis dissimilarity on each of our samples. In each analysis, there was no distinct pattern of clustering; however, samples from the same patient tended to cluster closely together regardless of date, further suggesting that the salivary microbiome is not significantly perturbed by short-term hospitalization (Fig. 7B–D). PERMANOVA analysis confirmed that there were no significant differences between the samples on days zero and three for each distance metric (p value > 0.9, PERMANOVA). Similar results were found using the V4V5 region (Supplementary Figure S3). These observations suggest that not only is there little inter-individual variation within the salivary microbiome, but that this community is unaffected by short-term hospitalization. This observation also contrasts with previous findings using the gut microbiota, which is known to have a high level of inter-individual and temporal variation^34, 59, 64^. The lack of significant differences after short-term hospitalization could also suggest that the salivary microbiome cannot be perturbed at all, or that our techniques are not sufficiently sensitive to detect these changes. To address this concern, we collected saliva samples from patients undergoing therapy with azithromycin, a narrow-spectrum macrolide antibiotic known to induce changes in the gut microbiome^65^. By doing so, we hoped that these communities would verify our capabilities to detect perturbations within the salivary microbiome. While short-term azithromycin did not induce significant community shifts in beta diversity, we observed a significant reduction in both Shannon (paired t-test: p = 0.0334) and Simpson diversity (paired t-test: p = 0.0483) after three days of azithromycin treatment (Supplementary Figure S4). Additionally, we were able to detect statistically significant increase of *Firmicutes* from 45.1% to 53.7% (negative binomial Wald test: p = 0.045) and reduction of *Fusobacteria* from 4.95% to 1.72% (negative binomial Wald test: p = 0.034) during therapy (Supplementary Figure S4). These data demonstrate that, unlike short-term hospitalization, clinical azithromycin treatment does impact the salivary microbiome. This is an interesting and novel finding, indicating that it is possible to induce perturbations in the salivary microbiome and that our pipeline is sensitive enough to detect these changes. Therefore, we feel that these observations strengthen our central finding that short-term hospitalization does not significantly perturb the salivary microbiome.

In conclusion, we present a streamlined workflow for the analysis of the salivary microbiome. We validated our extraction, sequencing, and analysis workflows using mock microbial communities available publicly through BEI Resources. To further validate our pipeline, we developed our own oral-specific mock community to test our ability to detect key taxa within this niche. Using these resources, we compared two commonly sequenced regions (V1V2 and V4V5) to determine which was better suited for the analysis of the salivary microbiome. Though both regions have distinct strengths and weaknesses, we concluded that the V1V2 region was more appropriate for general analysis of the salivary microbiome. Using our pipeline, we demonstrate that there is comparatively little inter-individual variation in the composition of the salivary microbiome, a finding that contrasts sharply with what has been previously reported using the gut microbiota^34^.

Additionally, we demonstrate that the salivary microbiome is largely unaffected by short-term hospitalization, suggesting that these communities are temporally stable during the timeframe that we tested. However, we did not explore the possibility that long-term hospitalization may have a more dramatic effect on the salivary microbiome; therefore, more research is required on this topic in the future. This finding differs dramatically from what has been observed in the gut, where short-term hospitalization is associated with dramatic taxonomic shifts that may increase risk of *C. difficile*-induced colitis^39, 42–44^. Furthermore, this observation is particularly noteworthy because our study participants varied with respect to age and diagnosis. Previous literature has demonstrated that the gut microbiota changes with both age^66^ and systemic pathologies^67^. Therefore, the fact that we detected no significant changes in the salivary microbiome after short-term hospitalization is a testament to the baseline stability of this community. One possible limitation intrinsic to this clinical study is that our sample size may have been insufficient to detect small changes in microbial population dynamics. However, we were able to detect changes in the salivary microbiome in response to short-term narrow spectrum antibiotic treatment with the same sample size. Despite the need for future expanded analysis, our data suggest that the salivary microbiome is remarkably stable while still being capable of being perturbed by clinical intervention. Though more research is needed on this topic, these initial findings suggest that the salivary community may be less susceptible to dysbiosis during short-term hospitalization.

## Materials and Methods

### Sample Collection

All collection procedures were approved and performed in accordance with the policies of Brown University, Rhode Island Hospital, and the Rhode Island Hospital Institutional Review Board. All study participants provided written informed consent. Saliva samples were collected from participants admitted to Rhode Island Hospital or receiving treatment at The Miriam Hospital clinic. Individuals who received antibiotic or immunosuppressive therapy within the six months prior to admission or during their admission were excluded from this study. For at least 30 minutes prior to sample collection, participants were asked to refrain from eating, drinking, smoking, or dental hygiene procedures (such as brushing and flossing). One milliliter of unstimulated saliva was collected from each patient using the OMNIgene-Oral kit (OM-505) (DNA Genotek, Ontario Canada) upon admission (Day 1) and three days later (Day 4) at approximately the same time on both days. Samples were then stored within 30 minutes at −80 °C until DNA extraction.

### Nucleic Acid Extraction

Microbial DNA and RNA were extracted from 48 total saliva samples from 24 individuals. To initiate lysis of microbial populations, samples were first incubated at 50 °C for one hour followed by a fifteen-minute incubation at 75 °C in OMNIgene-Oral kit buffer. Bacterial populations were then mechanically and enzymatically disrupted using lysozyme, proteinase K, and bead bashing. DNA and RNA were purified in parallel using the RNA Clean & Concentrator™-5 Kit (Zymo Research, Irvine, CA, U.S.) according to manufacturer’s instructions. DNA and RNA concentrations were quantified using the Qubit™ 3.0 Fluorometer with the dsDNA-HS and RNA-HS kits (Thermo Fisher Scientific, Waltham, MA, U.S.) according to manufacturer’s protocols.

### PCR Amplification

Both the V1V2 and V4V5 regions of the bacterial 16S rRNA gene were amplified using fusion primers with partial Illumina adaptors. To amplify V4V5, we utilized the universal bacterial 518F^68^ forward primer (CCAGCAGCYGCGGTAAN) and a mixture of three 926R^69^ reverse primers (CCGTCAATTCNTTTRAGT; CCGTCAATTTCTTTGAGT; CCGTCTATTCCTTTGANT) in an 8:1:1 ratio. Primer sequences and mixing ratios were obtained from the Marine Biological Laboratory (https://vamps.mbl.edu/resources/primers.php). The V1V2 region was amplified using primers 27F^70^ (AGAGTTTGATCMTGGCTCAG) and 338R^71^ (GCTGCCTCCCGTAGGAGT). The full sequences of all oligonucleotides used during this study are found in Supplementary Table S5. PCR reactions were prepared in 100 µL reaction volumes containing 20 µL of 5X Phusion® High-Fidelity (HF) buffer, 2 µL of 10 mM dNTPs, 1 µL of each primer (50 µM), 0.5 µL of Phusion® High-Fidelity DNA Polymerase, and 10 ng of template DNA. Master mixes were then split into triplicate reactions of 33 µL and amplified separately. Both the V1V2 and V4V5 regions of the bacterial 16S rRNA gene were amplified using PCR (98 °C for 30 seconds, followed by 25 cycles at 98 °C for 10 seconds, 57 °C for 30 seconds and 72 °C for 30 seconds, and a final extension at 72 °C for 5 minutes).

### Illumina MiSeq Sequencing

Following amplification, triplicate samples were pooled and then run on a 2% agarose gel to verify that the amplicons were the correct size (~450–500 bp). Samples were then submitted to the Rhode Island Genomics and Sequencing Center at the University of Rhode Island (Kingston, RI, USA) for indexing, quality control, and sequencing. Amplicons were paired-end sequenced (2 × 250 bp) on an Illumina MiSeq platform using a 500-cycle kit using standard protocols.

### Processing of Sequencing Data

Raw FASTQ reads were quality filtered, trimmed, de-noised, and merged using the DADA2 package^53^ (version 1.2.2) in R (version 3.3.1). Taxonomy was assigned to all ribosomal sequence variants using the RDP Classifier algorithm^72^ with RDP Training Set 14 (rdp_train_set_14) via the assignTaxonomy function in DADA2.

### Human Microbiome Project Mock Communities

The following reagent was obtained through BEI Resources, NIAID, NIH as part of the Human Microbiome Project: Genomic DNA from Mock Microbial Community B (Staggered, Low Concentration), v5.2 L, for 16S rRNA Gene Sequencing (HM-783D). 16S amplicons were then prepared for sequencing as described previously.

### Quantitative PCR (qPCR) and Oral Mock Community Generation

Genomic DNA from *Prevotella melaninogenica, Veillonella sp. 3_1_44, Actinomyces odontolyticus, Neisseria* Oral Taxon 014, *Streptococcus salivarius, Porphyromonas uenonis, Gemella morbillorum, Lactobacillus crispatus, Fusobacterium periodonticum, Oribacterium sinus*, and *Campylobacter upsaliensis* were obtained from BEI Resources (Manassas, VA, USA).

16S copy number quantifications for generation for each source of genomic DNA were determined via quantitative PCR. 16S rRNA gene amplicons were generated using universal primers (Supplementary Table S5)^73, 74^ and the SYBR-green based Essential DNA Green Start Master Mix (Roche, Indianapolis, IN, USA). Samples were amplified on the Roche Light Cycler™ 96 system. Direct quantification of 16S copy number was acquired by generating a standard curve that references a cloned bacterial DNA fragment containing 179 base pairs of the 16S rRNA gene^73^. Genomic DNA was subsequently diluted and mixed in various ratios to produce even and staggered mock communities (Supplementary Tables S3 and S4).

### Calculation of Diversity Metrics

Rarefaction curves were generated by subsampling evenly to a depth of 27,000 reads in increments of 500. The average number of unique ribosomal sequence variants detected at each sequencing depth was calculated to determine mean richness for each region tested. Shannon and Simpson diversity indices were calculated using the estimate_richness() function within the phyloseq (version 1.19.1) package in R. Alpha diversity metrics were averaged and plotted using Prism (version 6.0).

### Statistical Analyses

All statistical analyses were conducted in R (Version 3.3.2) using the vegan (version 2.4.2) and phyloseq (version 1.19.1) packages. Testing for differential abundance was conducted using a nonparametric Wald negative binomial test available within the DESeq. 2 (Version 1.14.1) package in R. P values were subsequently adjusted for multiple comparisons using the Benjamini and Hochberg method^75^. A PERMANOVA test was performed on beta diversity distance matrices using the adonis() function in the vegan package with 999 permutations.

### Data Availability

Raw reads were deposited into the NCBI Sequence Read Archive (SRA) database under the BioProject ID number PRJNA380250.

## Electronic supplementary material

Supplementary Figures
