# Supplementary material for: The salivary microbiome is consistent between subjects and resistant to impacts of short-term hospitalization
Source: Sci Rep. 2017 Sep 8;7:11040. doi: 10.1038/s41598-017-11427-2 (PMC5591268; doi:10.1038/s41598-017-11427-2)
Supplement: Supplementary file 1 — Supplementary Figures [file 41598_2017_11427_MOESM1_ESM.pdf]

# The salivary microbiome is consistent between subjects and resistant to impacts of short-term hospitalization

Damien J. Cabral[1], Jenna I. Wurster[1], Myrto E. Flokas[2], Michail Alevizakos[2], Michelle Zabat[1], Benjamin J. Korry[1], Aislinn D. Rowan[1], William H. Sano[1], Nikolaos Andreatos, MD[2], R. Bobby Ducharme, BA[3], Philip A. Chan, MD, MS[3], Eleftherios Mylonakis, MD, PhD[2], Beth Burgwyn Fuchs[2], Peter Belenky[1*]

[1] Department of Molecular Microbiology and Immunology, Division of Biology and Medicine, Brown University, Providence, RI 02912

[2] Division of Infectious Diseases, Rhode Island Hospital, Alpert Medical School and Brown University, Providence, RI 02903

[3] Department of Medicine, Brown University, Providence, RI 02903

[*] Peter_Belenky@brown.edu

| Organism | NCBI Reference Sequence | Theoretical rRNA Operons |
|---|---|---|
| ***Acintobacter baumannii*** | NC_009085 | 10,000 |
| ***Actinomyces odontolyticus*** | NZ_DS264586, NZ_DS264585 | 1,000 |
| ***Bacillus cereus*** | NC_003909 | 100,000 |
| ***Bacteroides vulgatus*** | NC_009614 | 1,000 |
| ***Clostridium beijerinckii*** | NC_009617 | 100,000 |
| ***Deinococcus radiodurans*** | NC_001263, NC_001264 | 1,000 |
| ***Enterococcus faecalis*** | NC_17316 | 1,000 |
| ***Escherichia coli*** | NC_000913 | 1,000,000 |
| ***Helicobacter pylori*** | NC_000915 | 10,000 |
| ***Lactobacillus gasseri*** | NC_008530 | 10,000 |
| ***Listeria monocytogenes*** | NC_003210 | 10,000 |
| ***Neisseria meningitidis*** | NC_003112 | 10,000 |
| ***Propionibacterium acnes*** | NC_006085 | 10,000 |
| ***Pseudomonas aeruginosa*** | NC_002516 | 100,000 |
| ***Rhodobacter sphaeroides*** | NC_007493, NC_007494 | 1,000,000 |
| ***Staphylococcus aureus*** | NZ_AASB00000000 | 100,000 |
| ***Staphylococcus epidermidis*** | NC_004461 | 1,000,000 |
| ***Streptococcus agalactiae*** | NC_004116 | 100,000 |
| ***Streptococcus mutans*** | NC_004350 | 1,000,000 |
| ***Streptococcus pneumonia*** | NC_003028 | 1,000 |

**Supplementary Table S1:** Composition of HMP Microbial Mock Community HM-783D

| Genus | Theoretical Relative Abundance (%) | Observed Relative Abundance with V1V2 (%) | Observed Relative Abundance with V4V5 (%) |
|---|---|---|---|
| ***Acintobacter*** | 0.21905805 | 0.316695 | 0.2034828 |
| ***Actinomyces*** | 0.021905805 | 0.03497826 | 0 |
| ***Bacillus*** | 2.190580504 | 2.058045 | 0 |
| ***Bacteroides*** | 0.021905805 | 0.08035546 | 0.1563605 |
| ***Clostridium*** | 2.190580504 | 2.149745 | 2.501767 |
| ***Deinococcus*** | 0.021905805 | 0.01323502 | 0.06639964 |
| ***Enterococcus*** | 0.021905805 | 0.01796181 | 0 |
| ***Escherichia*** | 21.90580504 | 12.9429 | 14.48155 |
| ***Helicobacter*** | 0.21905805 | 0.3838155 | 0 |
| ***Lactobacillus*** | 0.21905805 | 0.2788807 | 0 |
| ***Listeria*** | 0.21905805 | 0.1673284 | 0 |
| ***Neisseria*** | 0.21905805 | 0.3507279 | 0.4712233 |
| ***Propionibacterium*** | 0.21905805 | 0.25052 | 0 |
| ***Pseudomonas*** | 2.190580504 | 1.227075 | 0.7753764 |
| ***Rhodobacter*** | 21.90580504 | 10.88675 | 17.34958 |
| ***Staphylococcus*** | 24.09638554 | 27.96559 | 0.8203569 |
| ***Streptococcus*** | 24.11829135 | 35.52845 | 60.96986 |

**Supplementary Table S2:** Theoretical and Observed Relative Abundances for Microbial Mock Community HM-783D

| Organism | BEI Catalog Number | Theoretical 16S Operons | Theoretical Relative Abundance (%) | Observed Relative Abundance with V1V2 (%) | Observed Relative Abundance with V4V5 (%) |
|---|---|---|---|---|---|
| ***Prevotella melaninogenica*** | HM-80D | 100000 | 9.090909091 | 3.179866352 | 5.534897789 |
| ***Veillonella sp.*** | HM-64D | 100000 | 9.090909091 | 13.24542013 | 11.50746348 |
| ***Actinomyces odontolyticus*** | HM-94D | 100000 | 9.090909091 | 11.77173057 | 5.55802658 |
| ***Neisseria sp.*** | HM-91D | 100000 | 9.090909091 | 18.18100973 | 35.16465921 |
| ***Streptococcus salivarius*** | HM-121D | 100000 | 9.090909091 | 6.244124298 | 5.650541747 |
| ***Porphyromonas uenonis*** | HM-130D | 100000 | 9.090909091 | 9.764463755 | 0.02490793 |
| ***Gemella morbillorum*** | HM-240D | 100000 | 9.090909091 | 27.5770511 | 28.29007063 |
| ***Lactobacillus crispatus*** | HM-370D | 100000 | 9.090909091 | 4.125060345 | 4.449623712 |
| ***Fusobacterium periodonticum*** | HM-41D | 100000 | 9.090909091 | 0.833396854 | 1.184905795 |
| ***Oribacterium sinus*** | HM-13D | 100000 | 9.090909091 | 0.589475824 | 1.023004252 |
| ***Campylobacter upsaliensis*** | HM-297D | 100000 | 9.090909091 | 4.455370074 | 1.59054922 |

**Supplementary Table S3:** Composition, and Theoretical and Observed Relative Abundances of Evenly Distributed Oral Mock Community

| Organism | BEI Catalog Number | Theoretical 16S Operons | Theoretical Relative Abundance (%) | Observed Relative Abundance with V1V2 (%) | Observed Relative Abundance with V4V5 (%) |
|---|---|---|---|---|---|
| ***Prevotella melaninogenica*** | HM-80D | 1000000 | 22.6 | 16.32212415 | 23.69602535 |
| ***Veillonella sp.*** | HM-64D | 10000 | 0.226 | 0.348676052 | 0.347726572 |
| ***Actinomyces odontolyticus*** | HM-94D | 1000000 | 22.6 | 45.59632358 | 23.46714204 |
| ***Neisseria sp.*** | HM-91D | 100000 | 2.26 | 8.732949157 | 18.12799859 |
| ***Streptococcus salivarius*** | HM-121D | 100000 | 2.26 | 1.594572908 | 1.837668911 |
| ***Porphyromonas uenonis*** | HM-130D | 100000 | 2.26 | 2.892990007 | 0.013204807 |
| ***Gemella morbillorum*** | HM-240D | 10000 | 0.226 | 0.528120213 | 1.045380519 |
| ***Lactobacillus crispatus*** | HM-370D | 1000000 | 22.6 | 21.6295864 | 28.47836613 |
| ***Fusobacterium periodonticum*** | HM-41D | 100000 | 2.26 | 0.239258881 | 0.358730578 |
| ***Oribacterium sinus*** | HM-13D | 1000000 | 22.6 | 2.001604785 | 2.590342885 |
| ***Campylobacter upsaliensis*** | HM-297D | 10000 | 0.226 | 0.100663798 | 0.037413619 |

**Supplementary Table S4:** Composition, and Theoretical and Observed Relative Abundances of Staggered Oral Mock Community

| Name | Sequences (5’ →3’) |
|---|---|
| 518F + overhang adapter | TCGTCGGCAGCGTCAGATGTGTATAAGAGACAG**CCAGCAGCYGCGGTAAN** |
| 926R + overhang adapter_1 | GTCTCGTGGGCTCGGAGATGTGTATAAGAGACAG**CCGTCAATTCNTTTRAGT** |
| 926R + overhang adapter_2 | GTCTCGTGGGCTCGGAGATGTGTATAAGAGACAG**CCGTCAATTTCTTTGAGT** |
| 926R + overhang adapter_3 | GTCTCGTGGGCTCGGAGATGTGTATAAGAGACAG**CCGTCTATTCCTTTGANT** |
| 27F + overhang adapter | TCGTCGGCAGCGTCAGATGTGTATAAGAGACAG**AGAGTTTGATCMTGGCTCAG** |
| 338R + overhang adapter | GTCTCGTGGGCTCGGAGATGTGTATAAGAGACAG**GCTGCCTCCCGTAGGAGT** |
| Eubacteria_16S_qPCR_FWD | ACTCCTACGGGAGGCAGCAGT |
| Eubacteria_16S_qPCR_REV | ATTACCGCGGCTGCTGGC |

**Supplementary Table S5:** Oligonucleotides used in this study. Bolded regions denote universal primer sequences. Underlined regions denote overhang adapters needed to add Nextera indices. Note: 926R primers were mixed in an 8:1:1 ratio, respectively.

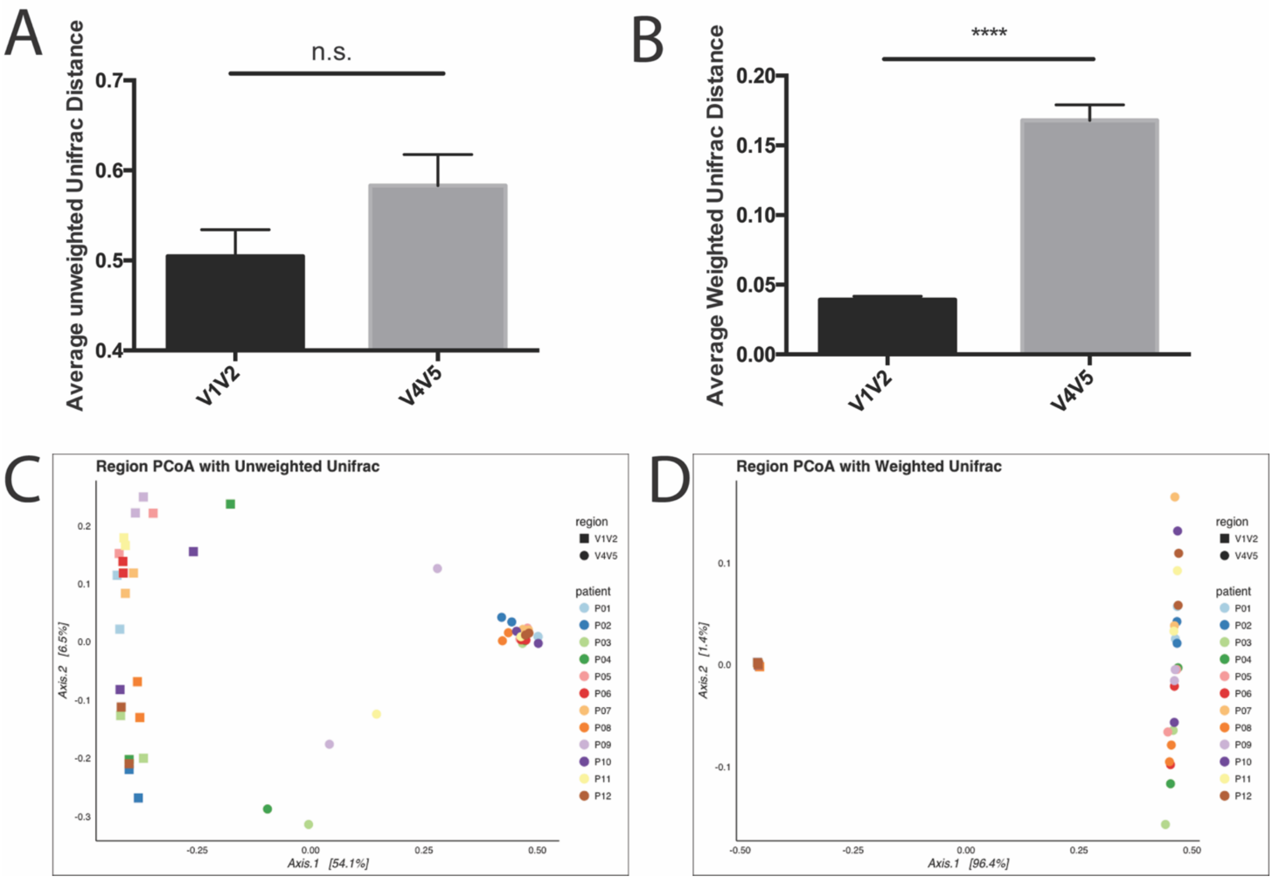


**Supplementary Figure S1: Comparison of V1V2 and V4V5 Hypervariable Regions using Various Beta Diversity Metrics** **(A)** Average unweighted Unifrac and (B) weighted Unifrac distances between all samples for both hypervariable regions. (C) PCoA analysis of the V1V2 and V4V5 hypervariable regions using unweighted and (D) weighted Unifrac

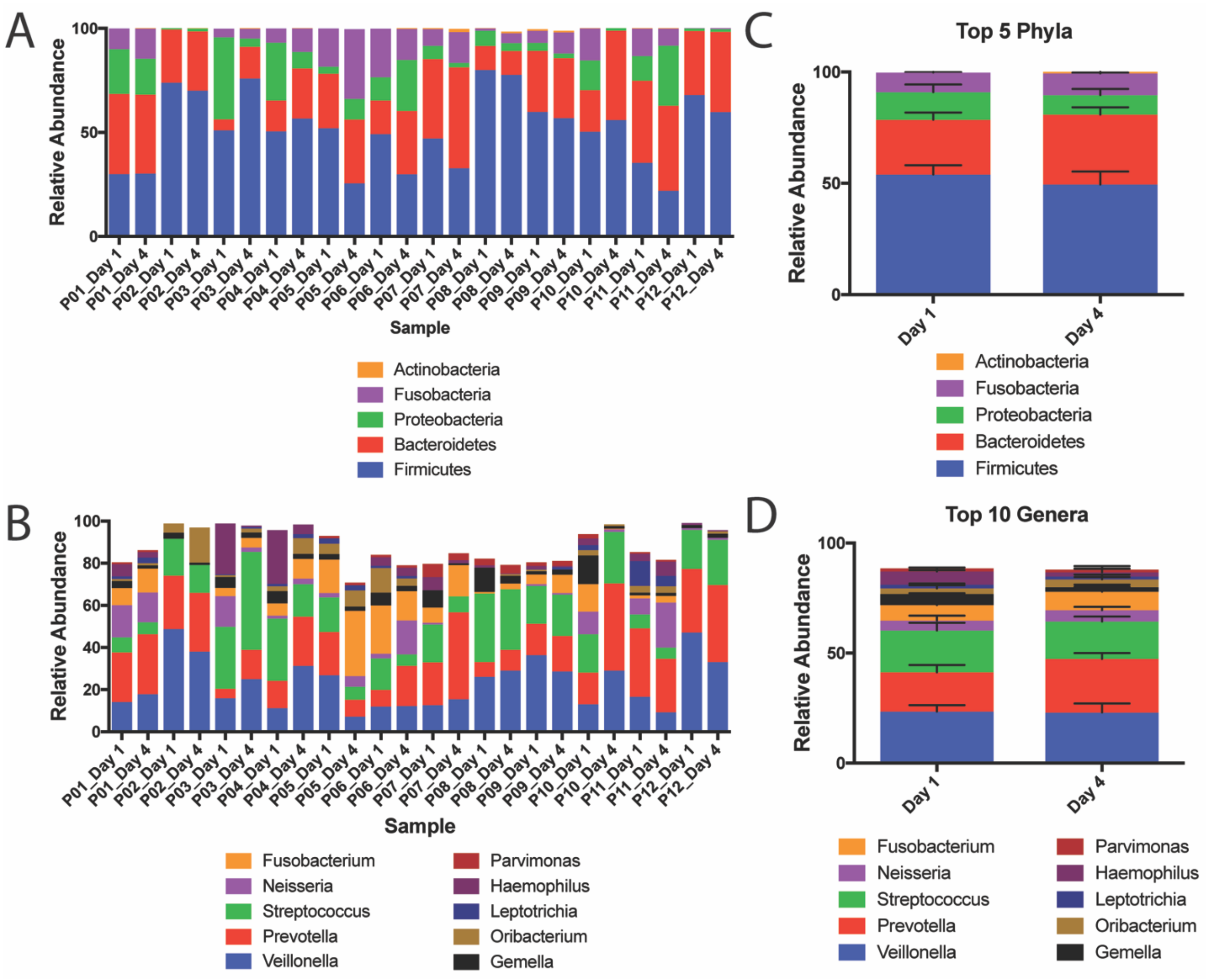


**Supplementary Figure S2: Taxonomic Analysis of Saliva Samples Before and After Hospitalization using V4V5**

(A) Relative abundances of the top five phyla detected in each sample using the V4V5 hypervariable region. (B) Relative abundances of the top ten genera detected in each sample using the V4V5 hypervariable region. (C) Average relative abundances of the top five detected phyla in all patient samples combined on days 1 and 4 of hospitalization. (D) Average relative abundances of the top ten detected genera in all patient samples combined on days 1 and 4 of hospitalization.

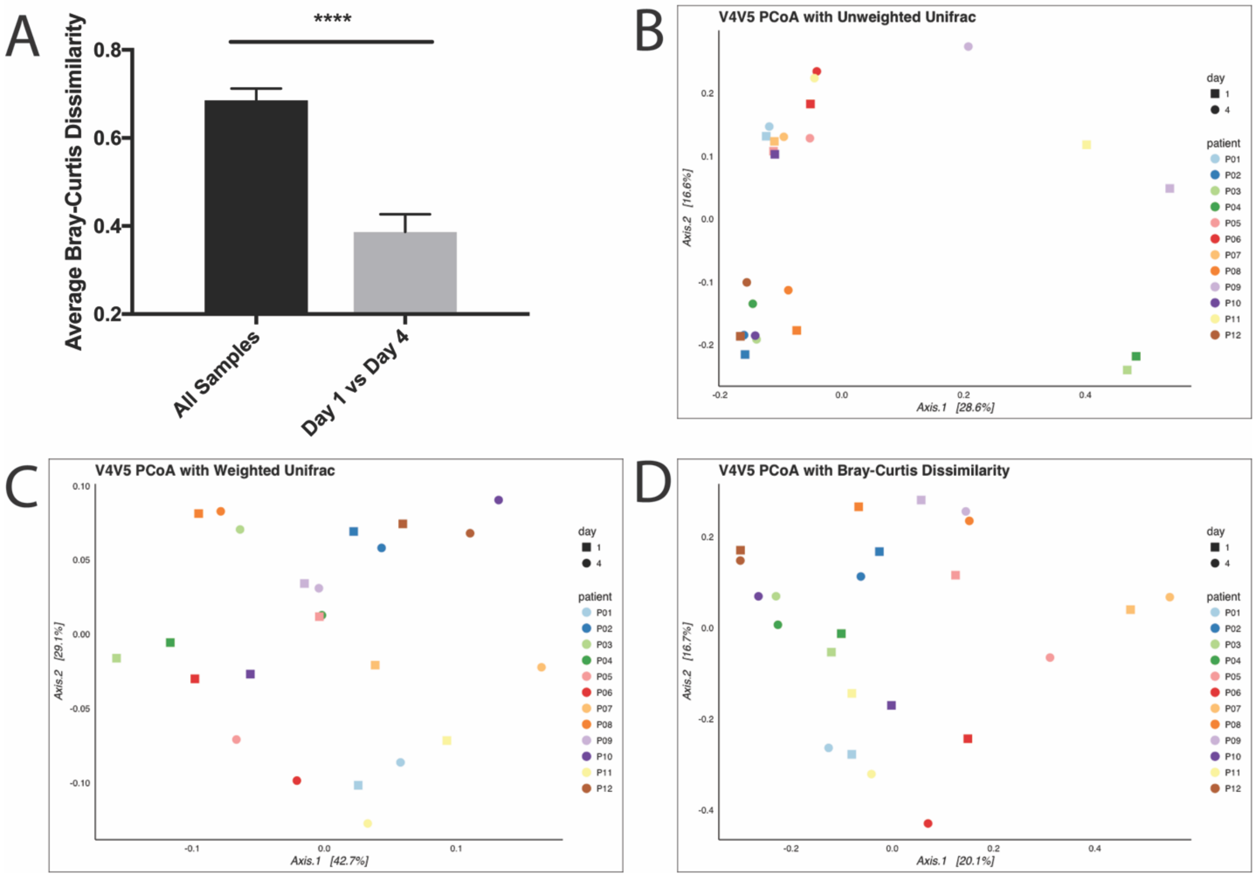


**Supplementary Figure S3: Beta Diversity Analysis of Saliva Samples Before and After Hospitalization Using V4V5**

(A)Average Bray-Curtis dissimilarity distances between all samples and between sample pairs before and after hospitalization (B-D) Principle Coordinate Analyses (PCoA) using unweighted Unifrac, weighted Unifrac and Bray-Curtis dissimilarity for patient samples on days 1 and 4.

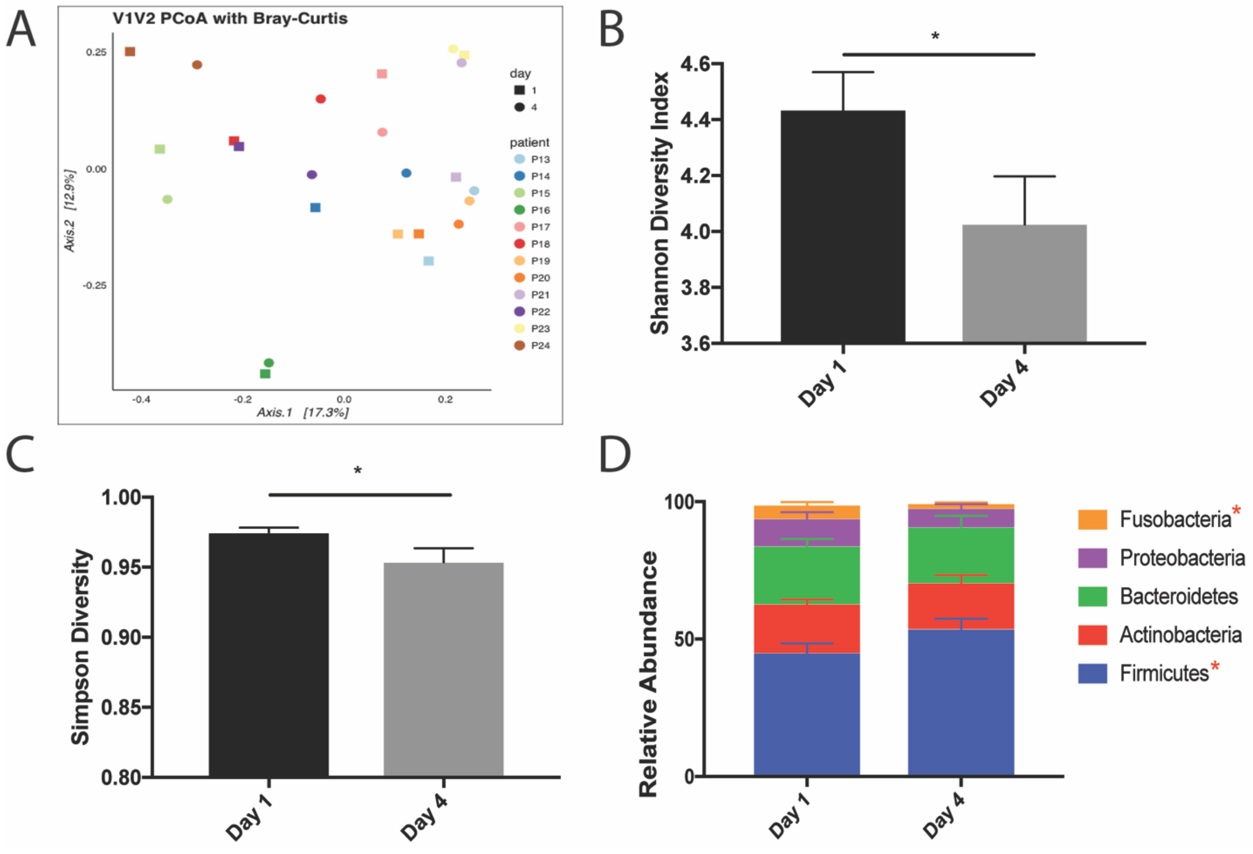


**Supplementary Figure S4: Narrow-Spectrum Antibiotic Therapy with Azithromycin Significantly Perturbs the Salivary Microbiome**

(A) PCoA Analysis using Bray-Curtis Dissimilarity of patients treated with azithromycin on day 1 and 4. (B) Average Shannon diversity and (C) Simpson diversity of patients treated with azithromycin on day 1 and 4. (D) Average relative abundances of the top five detected phyla in azithromycin-treated samples on days 1 and 4. Statistically significant phyla (negative binomial Wald test with Benjamini and Hochberg adjustment for multiple comparisons) are denoted with a red asterisk.
